# Quality of malaria services offered in public health facilities in three provinces of Mozambique: a cross-sectional study

**DOI:** 10.1186/s12936-019-2796-9

**Published:** 2019-05-06

**Authors:** Baltazar Candrinho, Mateusz M. Plucinski, James M. Colborn, Mariana da Silva, Guidion Mathe, Mercia Dimene, Ana Rita Chico, Ana Christina Castel-Branco, Frederico Brito, Marcel Andela, Gabriel Ponce de Leon, Abuchahama Saifodine, Rose Zulliger

**Affiliations:** 1 National Malaria Control Programme, Ministry of Health, Maputo, Mozambique; 2 United States President’s Malaria Initiative, Malaria Branch, Division of Parasitic Diseases and Malaria, United States Centers for Disease Control and Prevention, Atlanta, GA USA; 3 Clinton Health Access Initiative, Maputo, Mozambique; 4 UNICEF, Maputo, Mozambique; 5 United States President’s Malaria Initiative, United States Agency for International Development, Maputo, Mozambique; 6 United States President’s Malaria Initiative, United States Centers for Disease Control and Prevention, Maputo, Mozambique

**Keywords:** Health facility survey, Quality of care, Mozambique, Malaria case management

## Abstract

**Background:**

Fever associated with malaria is the leading cause of health care-seeking in Mozambique, yet there is limited evidence on the quality of malaria case management. This study evaluated the quality of malaria service provision offered in public health facilities in Mozambique.

**Methods:**

A cross-sectional assessment was conducted in April–May 2018 in three provinces of Mozambique: Maputo Province (low malaria burden), Cabo Delgado (high), and Zambézia (high). The study included all secondary and tertiary facilities and a random sample of primary facilities in each province. Data collection included exit interviews and re-examinations of 20 randomly selected outpatient service patients, interviews with up to five health care providers and the health facility director, a stockroom inventory and routine data abstraction.

**Results:**

A total of 319 health care providers and 1840 patients from 117 health facilities were included. Of these, 1325 patients (72%) had suspected malaria (fever/history of fever) and 550 (30%) had febrile, confirmed malaria with the highest burden in Cabo Delgado (43%), followed by Zambézia (34%) and Maputo Province (2%). Appropriate management of malaria cases, defined as testing malaria suspects and treating confirmed cases with the correct dose of anti-malarial, was highest in Zambézia and Cabo Delgado where 52% (95% CI 42–62) and 49% (42–57) of febrile malaria cases were appropriately managed, respectively. Only 14% (5–34) of febrile cases in Maputo Province were appropriately managed. The biggest gap in the malaria case management pathway was failure to test febrile patients, with only 46% of patients with this indication tested for malaria in Maputo Province. Additionally, anti-malarial treatment of patients with a negative malaria test result was common, ranging from 8% (2–23) in Maputo Province to 22% (14–32) of patients with a negative test in Zambézia. Only 58–62% of patients prescribed an anti-malarial correctly recited dosing instructions. Provider training and malaria knowledge was low outside of Zambézia and supervision rates were low in all provinces. Factors associated with correct case management varied by province and included patient age, facility type, treatment and testing availability, supervision, and training.

**Conclusion:**

These findings underscore the need to strengthen provider testing of all patients with fever, provider adherence to negative test results, and effective counselling of patients across epidemiological settings in Mozambique.

**Electronic supplementary material:**

The online version of this article (10.1186/s12936-019-2796-9) contains supplementary material, which is available to authorized users.

## Background

Malaria transmission occurs throughout Mozambique, ranging from low and seasonal transmission in the south to some of the world’s highest transmission rates in the holo-endemic centre and north of the country [1]. Malaria control in Mozambique is based on the pillars of prevention, including vector control and prevention of malaria in pregnancy, prompt diagnosis and provision of treatment with efficacious anti-malarials and a strong malaria case surveillance system. Malaria care is delivered primarily through public health facilities and a network of community health workers (CHWs). In 2008, the National Malaria Control Programme (NMCP) rolled out a policy of evaluating all malaria suspects (defined as individuals with malaria symptoms such as fever, headache, tiredness, sweating, chills, muscle pains and general malaise) with either microscopy or rapid diagnostic tests (RDTs); in 2017, case management guidelines were revised to explicitly recommend malaria testing of all febrile patients [2]. A cascade training on these new guidelines was rolled out through most of the country beginning with Zambézia in mid-2018, but by April 2019 had not reached all provinces.

In Mozambique, malaria testing is done using RDTs that are available at all levels, including at the community, or microscopy, which is limited to hospitals and certain health centres with laboratory capacity. First-line treatment in public health facilities is artemether-lumefantrine (AL) for uncomplicated malaria cases, oral quinine for pregnant women in their first trimester, and injectable artesunate for all severe cases. AL and pre-referral rectal artesunate are used for treatment of cases at the community level. All four age/weight formulations of AL should be available at all levels [2]. Malaria commodities are part of the essential commodities that flow through a pull-based system in public health facilities and all malaria services are provided free of charge. Nevertheless, stock-outs at the facility level remain a perennial challenge.

All-cause, under-five child mortality in Mozambique has fallen substantially in recent years, declining from 233 deaths per 1000 live births in 1990 to 97 in 2011 [3, 4]. Moreover, household survey data have shown substantial progress in the proportion of children with fever who received a finger or heel prick (from 56% in 2011 [4] to 69% in 2018 [5]) and in the proportion of children receiving an anti-malarial who received artemisinin-based combination therapy (ACT) (from 60% in 2011 [4] to 99% in 2018 [5]). Additionally, the number of individuals dying from malaria in public health facilities has consistently declined over the years from 3245 in 2014 to 968 in 2018 [6, 7].

In addition to periodic household surveys, the NMCP monitors the provision of malaria care through analysis of routine, monthly data submitted by health facilities and CHWs through the health management information system (HMIS). Analysis of these data show that 42% of persons presenting for outpatient consultation in 2017 were tested for malaria (suspect cases) and that 24% had a positive malaria diagnosis (confirmed malaria cases).

Aggregation of individual case data is useful for monitoring malaria trends and identifying data quality issues, but it is not possible to conduct a comprehensive evaluation of case management practices solely using routine data. To complement routine monitoring, health facility surveys can rigorously evaluate malaria care delivery and have commonly been used by malaria control programs in various settings [8–24]. Researchers in Mozambique completed a retrospective analysis of routine patient register data in 2011, a few years after the introduction of RDTs and of AL; the analysis found that 72% of patients with negative RDT results received treatment [25]. Mozambique has subsequently provided additional national level training and supervision of health care providers, but little data exist to demonstrate whether case management has improved over time.

The NMCP in Mozambique implemented a health facility survey in 2018 to evaluate the quality of malaria case management and assess case management practices.

## Methods

### Study design

A cross-sectional survey of health facilities was performed in three provinces purposively selected due to their geographic and malaria burden diversity. At each health facility, survey teams assessed malaria stocks, interviewed healthcare workers (HCWs), performed exit interviews and re-examinations of outpatients, and compared routine data from register books and monthly reports.

### Study population, sample size, and study power

**Fig. 1:**
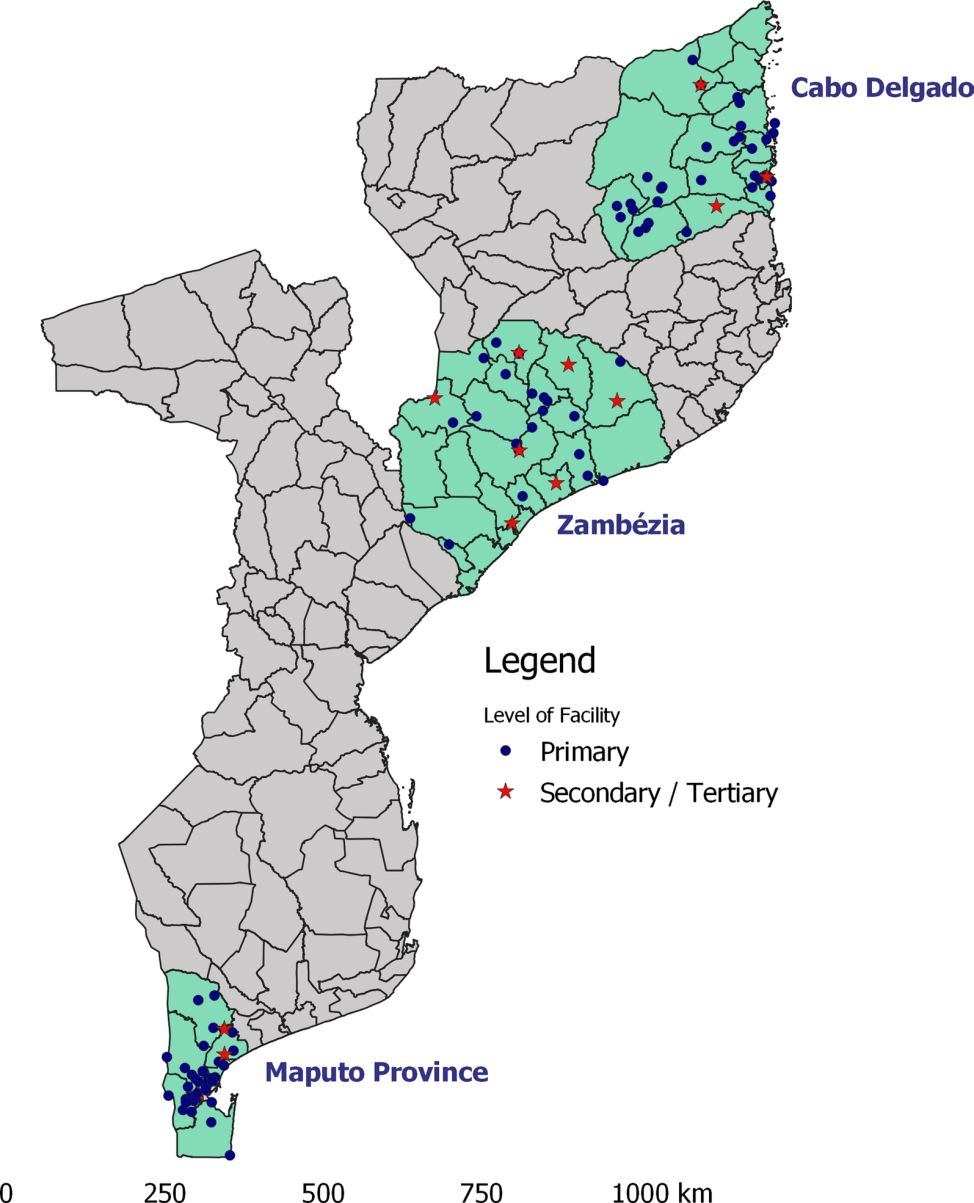
Location of health facilities visited as part of survey on malaria care delivery in Maputo, Zambézia, and Cabo Delgado Provinces, Mozambique, 2018

The survey was performed in low-transmission Maputo Province, and high-transmission Zambézia and Cabo Delgado Provinces (Fig. 1). These three provinces all have the same supply chain for malaria commodities, but at the time of the survey Zambézia was the only province that had implemented training on the new case management guidelines. All provinces have access to limited resources available for case management supervision, but Cabo Delgado and Zambézia have additional case management support for targeted health facilities through the President’s Malaria Initiative. Additionally, the three provinces have different burdens of malaria which has previously been shown to be associated with quality of malaria case management [8]. Sampling was stratified by province and by health facility type. In each province, all secondary and tertiary hospitals providing outpatient care in the province were exhaustively sampled, and then a random selection of primary health facilities was chosen to obtain a total of 40 health facilities per province. Health facilities that were closed or could not be accessed by survey teams were replaced by randomly selected health facilities. Three such health facilities were not replaced.

In each visited health facility, up to five HCWs engaged in outpatient care were conveniently chosen for inclusion in the survey. In addition, up to 20 outpatients (10 children and 10 adults) were randomly selected from all outpatients visiting the facility, regardless of symptoms, and invited to participate in the survey.

Assuming 20 outpatients would visit per health facility, per day, of whom 60% would be febrile, a test positivity rate of 40%, a rate of correct case management of 75%, and a design effect of 2, the survey was powered to estimate the proportion of true malaria cases correctly managed within ± 10 percentage points.

### Study period

In order to measure malaria case management practices when patient burden was at its highest, the survey was carried out in April to May 2018. This period coincided with both the end of the high malaria transmission and of the rainy season throughout the country.

### Data collection

Trained survey teams collected all study data in 1 day per selected health facility. HCW were interviewed using structured questionnaires (see Additional file 1) regarding their training and supervision and were administered a knowledge exam covering malaria diagnosis and treatment. The exam included questions related to RDT use, treatment administration, and management of malaria in pregnancy. Health facility directors were interviewed using structured questionnaires regarding availability of services, staff and resources. Survey teams performed an inventory of malaria commodities. This included the commodities for provision of malaria services at the health facility by HCWs and in communities by CHWs.

Outpatients selected for participation were interviewed using structured questionnaires following the completion of their health facility visit. They were asked to recall what symptoms they communicated to the HCW, whether or not the HCW had asked about fever or taken their temperature, what tests they had undergone, what medication was prescribed, and what treatment-related information was provided by the HCW. They also underwent a re-examination by a survey clinician, entailing a medical history, measurement of temperature (to determine if febrile), and testing for malaria with an HRP2-based *P. falciparum*-specific RDT (SD Bioline Pf, Yongin, Republic of Korea), independent of symptoms and whether or not they had been tested during the health facility consultation. Outpatients who did not have malaria symptoms were tested in order to better quantify the asymptomatic malaria reservoir in each province.

Data were collected on digital tablets using ODK-based Survey CTO software (Dobility, Cambridge, MA) and reviewed daily by team supervisors for quality purposes.

### Data analysis

Data were analysed using R version 3.3.2 (R Foundation for Statistical Computing, Vienna, Austria). Health facility readiness indicators, including availability of malaria commodities and HCW training and supervision, were calculated. Standard indicators for the quality of malaria case management were estimated from the exit interview and re-examination data. A suspect malaria case was defined as any patient reporting fever as a symptom of their current illness or with an axillary temperature ≥ 37.5 °C during the re-examination, and the proportion of suspect malaria cases tested for malaria during the health facility consult was calculated. Appropriate treatment was defined as oral quinine for pregnant women in the first trimester testing positive during re-examination, ACT for everyone else testing positive during re-examination, and no anti-malarial for anyone testing negative during re-examination. Appropriate management of a suspect malaria case was defined as testing by RDT or microscopy and treatment with the correct dose of the correct treatment, in accordance with the re-examination test result.

The subset of patients with febrile illness who tested positive by RDT during re-examination (symptomatic malaria cases) were further analysed along the full malaria case management pathway—identification of fever, testing, correct treatment, correct anti-malarial dose, and ability of the patient to recite the correct dosing schedule (as a proxy for receipt of effective malaria counseling). In addition, the frequencies of counseling practices for patients prescribed ACT were calculated. Effective malaria counseling was defined as patients being able to appropriately recall dosing schedule and how to take their treatment (e.g. with food, complete all doses, and seek care if symptoms persist).

All indicators were adjusted for the cluster-sampling design using the survey R package [26], weighting health facility-level observations by the probability of selection of each health facility, and the patient-level observations as the product of the health facility probability of selection and the patient-level probability of selection. The latter was calculated as the total number of patients interviewed in each health facility divided by the average patient flow for each health facility as reported through the HMIS for the month of the survey, separately calculated for patients under and those equal to and over 5 years of age.

A multivariate regression model was separately run for each province to investigate the relationship between correct management of suspect malaria cases, as defined above, and patient-, HCW-, and health facility-level variables.

### Ethical considerations

All interviewed HCWs, outpatients or their guardians provided written consent to be interviewed. The survey was reviewed and approved by the Mozambique National Health Bioethics Committee (338/CNBS/17) and the Office of the Associate Director for Science in the Center for Global Health at the Centers for Disease Control and Prevention (CGH2017-517).

## Results

A total of 117 facilities (39 in each province) were visited during the study, resulting in 1840 outpatient and 319 HCW interviews (Table 1). The majority (72%, 1325/1840) of surveyed outpatients presented with febrile illness, with fever rates ranging from 63% in Maputo to 78% in Zambézia (Table 1). After exclusion of fevers due to malaria, between 79 and 83% of children < 5 and 46–61% of older children and adults had non-malaria febrile illness. The proportion of febrile malaria infections ranged from 4% in Maputo to 42% in Zambézia. There were no afebrile RDT+ outpatients in Maputo, but 6% of patients in Zambézia and 3% in Cabo Delgado were afebrile but tested positive by RDT during the re-exam. Fever was the main motivator of care-seeking in 38% (95% Confidence Interval [CI] 31–44%) of patients in Maputo Province, 40% (32–49%) of patients in Zambézia, and 55% (47–64%) of patients in Cabo Delgado.

**Table 1:** Numbers and characteristics of health facilities, healthcare workers, and patients surveyed in Maputo, Zambézia, and Cabo Delgado Provinces, Mozambique, 2018

	n (%)			
	Maputo	Zambézia	Cabo Delgado	Total
Health facility	39	39	39	117
Primary	36 (92)	31 (79)	35 (90)	102 (87)
Secondary/tertiary	3 (8)	8 (21)	4 (10)	15 (13)
Healthcare workers interviewed	99	99	121	319
Patients interviewed	542	642	656	1840
< 5 years	154 (28)	228 (36)	247 (38)	629 (34)
5–15 years	120 (22)	87 (14)	101 (15)	308 (17)
> 15 years	268 (49)	327 (51)	308 (47)	903 (49)
Female	321 (59)	395 (62)	356 (54)	1072 (58)
Suspect malaria cases^a^	343 (63)	472 (74)	510 (78)	1325 (72)
Symptomatic malaria cases^b^	24 (4)	249 (39)	277 (42)	550 (30)
Asymptomatic malaria cases^c^	0 (0)	40 (6)	22 (3)	62 (3)

^a^Fever/history of fever

^b^Fever/history of fever and positive by rapid diagnostic test during survey re-examination

^c^No fever/history of fever and positive by rapid diagnostic test during survey re-examination

### Stock availability

Nearly all facilities in Zambézia (97%, 95% CI 84–99%) and Cabo Delgado (97%, 95% CI 88–99%) had either RDTs or microscopy available on the day of the survey visit, compared to 88% (95% CI 77–94%) in Maputo (Table 2). Availability of any formulation of ACT ranged from 87% (95% CI 75–93%) in Cabo Delgado to 100% in Zambézia. Availability of all four formulations of ACT was significantly lower, ranging from 11% (95% CI 5–24%) in Zambézia to 50% (95% CI 38–63%) in Maputo. Additionally, there were high rates of stock-outs of oral quinine tablets (range: 38–46%) and CHW malaria commodity kits in the health facilities (range: 56–76%) in each province. Availability of commodities used for prevention of malaria in pregnancy was better, with lower facility stock-outs of sulfadoxine-pyrimethamine (14–29%) and of long-lasting insecticidal nets (3–27%) in each province (see Additional file 2).

**Table 2:** Standard key indicators on health facility readiness for malaria care delivery, as assessed in health facility surveys in Maputo, Zambézia, and Cabo Delgado Provinces, Mozambique, 2018

	Maputo		Zambézia		Cabo Delgado	
	%	95% CI	%	95% CI	%	95% CI
Health facilities						
Offering any malaria diagnostic services	88	77–94	97	84–99	97	88–99
RDT	85	74–92	95	85–99	97	88–99
Malaria microscopy	21	13–34	25	14–40	26	16–38
With any formulation of ACT available on day of visit	97	88–99	100	*	87	75–93
With all four formulations of ACT available on day of visit	50	38–63	11	5–24	35	24–48
With at least one HCW trained on RDT use	29	18–44	89	75–96	36	24–49
With at least one HCW trained on malaria microscopy	13	6–26	20	10–35	26	17–39
With at least one HCW trained on malaria treatment	19	10–33	90	75–96	41	29–54
With guidelines on malaria case management	53	39–66	93	79–98	51	38–64
HCWs ever trained in malaria case management	26	19–35	83	72–90	28	20–38
HCWs supervised in last 6 months	18	12–26	20	13–30	43	35–51
Mean score on knowledge test (out of 100)	48	45–52	77	73–81	56	53–59

RDT, rapid diagnostic test; ACT, artemisinin-based combination therapy; HCW, healthcare worker; CI, confidence interval

* Confidence intervals undefined

### HCW training status

Indicators on reported HCW training on malaria service delivery were significantly higher in Zambézia (range: 89–93%) than in Maputo (range: 19–29%) and Cabo Delgado (range: 28–41%). The mean HCW knowledge score was also higher in Zambézia (77%, 95% CI 73–81) where there was recently a HCW malaria training for all health care providers relative to Maputo (48%, 95% CI 45–52%) and Cabo Delgado (56%, 95% CI 53–59%) (Table 2, Additional file 3) where the trainings had not yet occured. Supervision rates in the six months preceding the survey were lower in Maputo (18%, 95% CI 12–26%) and Zambézia (20%, 95% CI 13–30%) compared to Cabo Delgado (43%, 95% CI 35–51%).

### Appropriate case management

Overall, the proportion of suspect malaria cases correctly managed was 29% (95% CI 21–39%) in Maputo, 44% (95% CI 37–52%) in Zambézia, and 48% (95% CI 41–55%) in Cabo Delgado (Table 3). Between 1% (Zambézia) and 8% (Maputo) of all patients reported having had their temperatures measured during the clinic visit, with between 1% (Zambezia) and 9% (Maputo) of patients reporting fever having had their temperatures measured. The rate of malaria testing for febrile patients was significantly lower in Maputo (33%, 95% CI 24–43%) compared to Zambézia (62%, 95% CI 53–70%) and Cabo Delgado (69%, 95% CI 60–77%). The proportion of positive cases provided with appropriate treatment was very high in Zambézia (87%, 95% CI 77–93%) and Cabo Delgado (90%, 95% CI 82–94); the estimate for the proportion of positive cases provided with appropriate treatment in Maputo (54%, 20–84) was imprecise because of the small number of confirmed cases in that province. There was substantial anti-malarial treatment of malaria-negative cases, ranging from 8% (95% CI 2–23) in Maputo to 22% (95% CI 14–32%) in Zambézia.

**Table 3:** Standard key indicators on healthcare worker performance in malaria case management, as assessed in health facility surveys in Maputo, Zambézia, and Cabo Delgado Provinces, Mozambique, 2018

	Maputo		Zambézia		Cabo Delgado	
	%	95% CI	%	95% CI	%	95% CI
Suspect malaria cases receiving malaria test	33	24–43	62	53–70	69	60–77
< 5 years	34	21–51	70	60–79	68	54–79
RDT	29	15–49	66	53–77	67	54–79
Microscopy	5.1	0.9–24	4.1	1–12	0.4	0.1–1.6
≥ 5 years	32	21–46	53	42–64	71	59–81
RDT	24	13–40	51	40–62	68	55–78
Microscopy	11	5–24	3.9	2–9	7.1	3–16
Confirmed malaria cases treated with appropriate antimalarial	54	20–84	87	77–93	90	82–94
Suspect malaria cases negative for malaria* but treated with antimalarial	7.7	2–23	22	14–32	16	8–32
Suspect malaria cases not tested, treated with appropriate antimalarial	0	****	1.1	0.2–6.5	2.7	0.5–13
Suspect malaria cases managed correctly**	29	21–39	44	37–52	48	41–55
True malaria cases appropriately treated***	14	5–34	52	42–62	49	42–57

RDT, rapid diagnostic test; CI, confidence interval

* During re-examination

** Tested and treated with antimalarial with correct dose only if positive

*** Treated with first-line antimalarial with correct dose

**** CI not defined

**Fig. 2:**
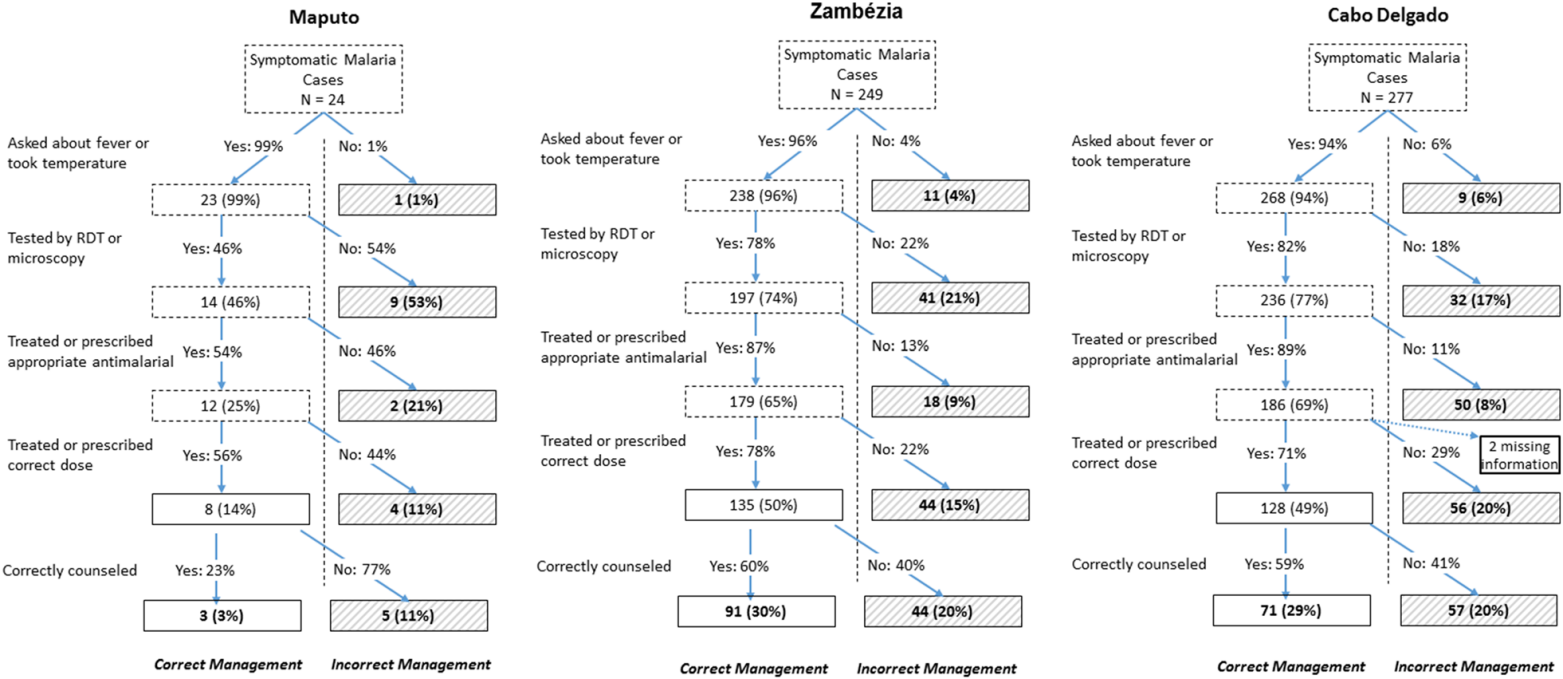
Malaria case management pathway for true malaria cases (as confirmed during re-exam) seen at health facilities in Maputo, Zambézia, and Cabo Delgado Provinces, Mozambique, 2018. Percentages in boxes outlined in dashed lines represent cumulative proportion of patients managed correctly to that point. Boxes outlined in bold denote final categorization and percentages refer to final proportion of cases falling into each final categorization. Percentages reflect adjustment for cluster-sampling design

When considering only patients with symptomatic malaria infection (reported fever or had fever during the re-examination) (Fig. 2), only 3% (95% CI 0.8–12%) of these were correctly managed along the entire malaria case management pathway in Maputo, compared to 30% (21–41%) in Zambézia and 29% (18–43%) in Cabo Delgado (Fig. 2). The most common error in Maputo and Zambézia not testing, with 53% of patients in Maputo and 21% of patients in Zambézia falling out of the correct pathway at this stage. In Cabo Delgado, most errors occurred at the step of choosing the correct dose for the ACT (20%) and providing correct counseling (20%), but there was also substantial under-testing (17%).

The effectiveness of counselling of patients prescribed ACT was low in all of the provinces (Table 4). A small minority of patients were given the first dose of the ACT during the consult (range: 7–8%), and only between 58 and 62% of patients were able to recite the correct dosing schedule. A minority of patients who were prescribed AL were counselled to take it with food or milk, ranging from 11% (95% CI 6–19%) in Zambézia to 40% (95% CI 22–61%) in Cabo Delgado. With the exception of Maputo, less than half of patients reported having been explicitly counselled to complete all doses or return if symptoms worsened or failed to improve.

**Table 4:** Quality of counseling in patients prescribed an ACT as assessed during exit interviews in health facility surveys in Maputo, Zambezia, and Cabo Delgado Provinces, Mozambique, 2018

	Maputo		Zambezia		Cabo Delgado	
		95% CI	0	95% CI	90	95% CI
Given first dose at health facility	7	2–21	8	4–16	7	3–14
Given instructions on how to take ACT	100	100–100	92	87–96	79	68–87
Able to correctly recite dosing schedule^a^	62	28–87	58	45–70	58	38–76
Received instructions to:						
Take AL with food or milk	18	3–57	11	6–19	40	22–61
Take AL on empty stomach	9	1–43	0	0.09–2.30	0	0.02–0.84
Complete all doses	72	34–93	42	31–55	38	20–60
Return if worse	66	30–90	38	29–48	40	22–61
Return if no improvement	18	5–49	39	29–50	38	21–60

ACT, artemisinin-based combination therapy; AL, artemether–lumefantrine; CI, confidence interval

^a^Correct number of tablets per dose, doses per day, and total duration of treatment. Calculated only for subset of patients prescribed the correct dose

### Factors associated with appropriate case management

In all three provinces, older suspect cases were less likely to be appropriately managed than children less than 5 years old, reaching statistical significance in Zambézia and Cabo Delgado (Table 5). In Maputo, primary health facilities were less likely to provide adequate malaria case management compared to secondary/tertiary hospitals (Odds ratio [OR]: 0.038, 95% CI 0.0082–0.14). Availability of RDTs (in Maputo) and ACTs (in Maputo and Cabo Delgado) was strongly associated with correct management of suspect cases, with ORs ranging from 2.4 to 10. Supervision of HCWs in Maputo (OR: 4.3, 95% CI 1.1–18) and Cabo Delgado (OR: 1.9, 95% CI 1.1–3.5) was associated with better case management, while HCW training was associated with better case management in Zambézia (OR: 3.2, 95% CI 1.3–7.8). The mean score of HCWs on the knowledge quiz within the health facility was not predictive of appropriate case management of patients attending that health facility in any of the provinces.

**Table 5:** Factors associated with correct management of suspect malaria cases attending health facilities in Maputo, Zambezia, and Cabo Delgado Provinces, Mozambique, 2015

Variable	Maputo		Zambia		Cabo Delgado	
	Adjusted odds ratio	95% CI	Adjusted odds ratio	95% CI	Adjusted odds ratio	95% CI
Patient age (years)						
<5	Ref	–	Ref	Ref		
5–15	1.1	0.57–2.2	1.1	0.62–2	1.1	0.66–2
>15	0.61	0.32–1.1	0.3	0.19–0.47	0.29	0.19–0.45
Patient sex						
Female	Ref	Ref	Ref			
Male	1.4	0.83–2.4	0.93	0.61–1.4	1.1	0.72–1.5
Health facility type						
Hospital	Ref	Ref	Ref			
Health center	0.04	0.008–0.14	0.87	0.5–1.5	0.93	0.49–1.8
RDT or microscopy available on day of visit	5.1	1.4–22	0.43	0.11–1.4		
ACT available on day of visit	10	1.6–208	2.4	1.2–5		
Proportion of interviewed HCWs supervised in last 6 months	4.3	1.1–18	0.47	0.2–1.1	1.9	1.1–3.5
Proportion of interviewed HCWs trained in malaria case management	0.55	0.16–1.8	3.2	1.3–7.8	0.87	0.41–1.8
Mean score on knowledge test of interviewed HCWs	1.1	0.1–12	0.3	0.069–1.3	4.7	0.81–28
Proportion of patients testing true positive by ROT during re-examination	144	0.75–29,540	0.78	0.19–3.2	0.49	0,19–1.3

RDT, rapid diagnostic test; ACT, artemisinin-based combination therapy; HCW, healthcare worker; CI, confidence interval

## Discussion

Despite making up the large majority of outpatients, less than half of suspect malaria cases were found to be appropriately managed for malaria in public health facilities in three provinces of Mozambique. Even after exclusion of fevers due to malaria, the majority of patients had a febrile illness, confirming studies showing fever to be highly prevalent in outpatients throughout sub-Saharan Africa [27]. Despite this preponderance of measured fever amongst outpatients, measuring temperatures was rare and malaria testing of fever cases was sub-optimal in all provinces, below 70% in high-transmission Zambézia and Cabo Delgado, and only 33% in low-transmission Maputo.

High availability of RDT or microscopy and high rates of identification of fever cases suggests that clinicians are making the decision to not test patients that they know have a febrile illness. Non-testing was the main contributor to inadequate management of malaria cases in Maputo and Zambézia, and an important contributor in Cabo Delgado. This reflects findings from previous health facility surveys in other settings that have shown the testing step in the malaria case management pathway to be a critical barrier to correct management [8, 9, 13, 18].

Case management of malaria is a complex multi-step process, and like other similarly complex interventions, requires high performance at each step to obtain high overall coverage [28]. The quality of malaria case management was particularly low in Maputo, where only 29% of suspect cases were correctly managed. Given that Maputo has the lowest transmission levels in the country [5], this low testing could reflect HCW perceptions of the risk of malaria, low rates of supervision and training, or gaps in HCW knowledge. This matches findings from other surveys showing lower rates of testing and correct case management in low-transmission areas [8]. This finding highlights the need for continued and intensified provider training on malaria case management as geographic areas transition from higher to lower malaria burden, particularly if regional elimination goals are to be met. Although overall malaria case management performance was better in Zambézia and Cabo Delgado, where malaria cases represent a higher proportion of all-cause consultations, there were still substantial weaknesses in malaria case management in these provinces. An additional gap in the management of fever was the treatment of malaria-negative cases with anti-malarials, which ranged from 8% to 22%. While it is alarming that a large proportion of patients are unnecessarily consuming ACT, this still demonstrates important improvements from 2011 when 72% of patients with negative RDT results received treatment [25].

Notably, these findings suggest counseling of patients prescribed ACT was not effective, with approximately 40% of patients in each province leaving the health facility not knowing how many tablets to take over how many days. Moreover, the majority of patients prescribed AL, the predominant artemisinin-based combination in use in Mozambique, did not recall being told of the need to take it with food or milk. Administration of AL with a fatty meal is crucial to maximize absorption of the lumefantrine component [29, 30], and not doing so risks inadequate clearance of parasitaemia and recrudescence. Additionally, failure to tell patients to immediately return to the health facility should symptoms not improve is a missed opportunity for improving malaria case outcomes. Thus, failure to appropriately counsel patients with malaria is an important gap in quality malaria case management. Factoring poor comprehension of the dosing schedule into the calculations of the coverage with adequate treatment in true malaria cases reduces the estimate of coverage to 30% or below in each province.

Importantly, many of the main conclusions drawn from this survey can be determined through analysis of the routine data. For example, the substantial under-testing in Maputo documented during the survey is consistent with the data reported by the province through the HMIS. In 2017, Maputo health facilities reported suspect cases to be 20% of all-cause patient consults. Because data from exit interviews showed that 63% of outpatients in Maputo present with fever, a rough upper bound for the testing rate of febrile cases is 32%, which matches the testing rate of 33% observed during this survey. This finding suggests that routine data can be used to make useful inferences about testing rates with high frequency and granularity [27]. There is, nevertheless, still value in periodic health facility surveys to complement routine data analysis in order to evaluate essential aspects of appropriate case management such as quality of patient counseling, non-testing of fevers, and appropriate treatment dispensing.

Limitations related to this study design may have affected the study results and their generalizability. Exit interviews minimize the risk of bias due to the Hawthorne effect, but are subject to bias due to patient recall [31]. Some of the findings of substandard counselling quality could be due to poor recall or inadequate understanding of the survey questions. Additionally, the cross-sectional design of the survey precludes extrapolation of the findings to other time periods, and it is not known how the case management practices documented here would change later in the transmission season when malaria risk is lower or in reaction to changes in commodity availability. Lastly, this study was designed to determine the quality of malaria case management for the general outpatient population and, as such, does not provide insights into the quality of case management for severe malaria or for important sub-groups such as pregnant women as only 17 pregnant women were included in the random selection. These are important areas for future investigation to better guide malaria case management supervision, training and commodity supply systems.

Nevertheless, the results of the risk factor analysis suggest that malaria case management would improve with better availability of commodities and additional trainings and supervisions, as each of these were significantly associated with appropriate malaria case management in the risk factor analysis. Future trainings might benefit from an increased focus on universal testing of all fever cases and better counselling. However, trainings should be complemented by other activities as malaria case management and counselling was poor even in Zambézia, which had high rates of HCWs who had undergone trainings. This underscores the importance of combining training with other interventions [32] such as continued supervision post-training to reinforce implementation of best practices. This supervision would benefit from a patient observation component, where testing and counselling practices can be directly and systematically assessed.

## Conclusion

This study of the quality of malaria case management in Mozambique found that while there have been important improvements made over time, critical gaps remain along the malaria case management cascade in Mozambique. Results varied across the different provinces included in this study, but failure to test all febrile patients and provide appropriate counseling was consistently problematic. These findings underscore the need to strengthen provider testing of all patients with fever, improve provider adherence to negative test results, and enhance effectiveness of counselling of patients across epidemiological settings in Mozambique.

## Additional files

**Additional file 1.** Mozambique Malaria Case Management Health Facility Survey Health Care Worker Questionnaire.

**Additional file 2.** Availability of malaria commodities during visits to health facilities in Maputo, Zambézia, and Cabo Delgado Provinces, Mozambique, 2018.

**Additional file 3.** Proportion of healthcare workers responding correctly to questions on malaria case management in Maputo, Zambézia, and Cabo Delgado Provinces, Mozambique, 2018.

## Abbreviations

AL: artemether–lumefantrine
ACT: artemisinin-based combination therapy
CHWs: community health workers
CI: confidence interval
HCWs: healthcare workers
HMIS: health management information system
NMCP: National Malaria Control Programme
OR: odds ratio
RDTs: rapid diagnostic tests
