# Supplementary material for: Quality of malaria services offered in public health facilities in three provinces of Mozambique: a cross-sectional study
Source: Malar J. 2019 May 6;18:162. doi: 10.1186/s12936-019-2796-9 (PMC6503352; doi:10.1186/s12936-019-2796-9)
Supplement: Supplementary file 1 — Additional file 1. Mozambique Malaria Case Management Health Facility Survey Health Care Worker Questionnaire. [file 12936_2019_2796_MOESM1_ESM.docx]

**Additional File 1.**

| **English** | **Portuguese** |
| --- | --- |
| Site Identification | Identificação do sítio |
| Province | Província |
| Site Code | Código da Unidade Sanitária |
| District Name | Nome do Distrito |
| Date | Data |
| Name of Health Facility | Nome da Unidade Sanitária |
|  |  |
| **Survey Team Information** | **Equipe de Inquiridor da Informação** |
| Name of Surveyor | Iniciais do inquiridor |
| Team Number | Número de Equipa |
|  |  |
| **Healthcare Worker Information** | **Informação demográfica do profissional de saúde** |
| Healthcare worker's age (years): | Idade do profissional de saúde (em anos): |
| Healthcare worker's gender: Male / Female | Sexo do profissional de saúde: Homem / Mulher |
| Healthcare worker's cadre:   \| Doctor \| \| --- \| \| Medical technician \| \| Nurse - Basic Education \| \| Nurse - High School \| \| Nurse - Higher Education \| \| Technical - Laboratory \| \| Pharmaceutical \| \| Pharmaceutical technician \| \| Birth attendant \| \| Other \| | Qual é a formação do profissional de saúde?   \| Médico(a) \| \| --- \| \| Técnico(a) de medicina \| \| Enfermeiro(a) ensino básico \| \| Enfermeiro(a) ensino médio \| \| Enfermeiro(a) ensino superior \| \| Técnico(a) de laboratório \| \| Farmacêutico \| \| Técnico de farmácia \| \| Parteira \| \| Outro \| |
| If Other, please specify: | Se outro, descreve |
|  |  |
| **Healthcare Worker Education** | **Formação do profissional de saúde** |
| How many years of health professional training do you have? | Você fez quantos anos de formação em saúde? |
| How many years have you been working? | Há quantos anos que você esta a trabalhar? |
| How many years have you been working in this health facility? | Há quantos anos esta a trabalhar nesta US? |
| How many years of experience do you have in providing malaria services? | Tem quantos anos de experiência na prestação dos serviços da malária? |
| What is your role in this health facility?  Health facility director  Head of outpatient services  Head of child and maternal health services  Head of emergency services | Qual é o seu papel na US?  Responsável da unidade sanitária  Responsável das consultas externas  Responsável da SMI  Responsável banco socorros |
| If Other, please specify: | Se outro, descreve |
| How many years ago did you get your last degree, diploma or certificate | Há quantos anos terminou o curso mais recente? |
| How many years of experience do you have providing malaria services to patients? | Tem quantos anos de experiência na prestação dos serviços da malaria? |
| Are you the facility in-charge? (Yes / No) | Você é o chefe da unidade sanitária? (Sim / Não) |
|  |  |
| **Healthcare Worker Training** | **Formação do profissional de saúde** |
| Have you ever attended an in-service training on malaria case management? (Yes / No) | Assistiu alguma vez a uma formação sobre manejo de casos de malária? (Sim / Não) |
| How many trainings have you attended? | Quantas formações assistiu? |
| **Training Information** | **Detalhes da Formação** |
| When did you most recently attend a case management course? | Quando assistiu a formação? |
| Who led the course?  National Ministry of Health  Provincial Ministry of Health  District Ministry of Health  Health Facility  Partner (specify: )  Do not remember | Quem deu a formação?  Ministério da Saúde Nacional  DPS  DDS  Unidade Sanitária  Parceiro (especifique: )  Não recorda |
| How many days long was the in-service training? | A formação foi de quantos dias? |
| Did the training include training on use of RDTs? (Yes / No) | A formação incluiu uma componente sobre o uso de TDRs? (Sim / Não) |
| Did the in-service training provide training on the use of antimalarials? (Yes / No) | A formação incluiu uma componente sobre o tratamento com antimaláricos? (Sim / Não) |
| Which drug or drugs were you trained on?   \| Arteméter-lumefantrina \| \| --- \| \| Artesunato-amodiaquina \| \| dihidroartemisinina-piperaquina \| \| sulfadoxina-pirimetamina \| \| quinina comprimidos \| \| quinine injectável \| \| chloroquine \| \| primaquine \| \| artesunate injectável \| \| rectal artesunate \| \| intramuscular artemether \| \| Other \| | Quais antimaláricos?   \| Arteméter-lumefantrina \| \| --- \| \| Artesunato-amodiaquina \| \| dihidroartemisinina-piperaquina \| \| sulfadoxina-pirimetamina \| \| quinina comprimidos \| \| quinine injectável \| \| chloroquine \| \| primaquine \| \| artesunate injectável \| \| rectal artesunate \| \| intramuscular artemether \| \| Other \| |
| Did the in-service training include any clinical practice with real patients? (Yes / No) | A formação incluiu sessões prácticas com pacientes reais? (Sim / Não) |
| Did the in-service training include a follow-up visit from a supervisor to your job? (Yes / No) | A seguir a formação, será que recebeu uma visita de supervisão ligada à formação? (Sim / Não) |
|  |  |
| **Guidelines** | **Directrizes Nacionais** |
| Do you have access to a copy of the most recent national malaria treatment guidelines? (Yes / No) | Tem acesso às normas nacionais de tratamento da malária? (Sim / Não) |
| What year were they created? | Vem de que ano? |
| Do you have access to a copy of the national malaria RDT guidelines or other reference materials on RDTs? (Yes / No) | Tem acesso a uma bula / prospecto sobre uso de TDRs? (Sim / Não) |
| What year were they created? | Vem de que ano? |
| Who created the document?  Ministry of Health  PMI  Other partner (specify: )  Not sure | Quem desenvolvou o documento?  Ministério da Saúde  Iniciativa Presidencial dos Estados Unidos  Contra a Malaria  Outro Parceiro (especifique: )  Não sabe |
| Do you have access to a copy of the dosing schedule for Coartem / AL? (Yes / No) | Tem acesso ao guía da dosagem de arteméter lumefantrina (Coartem)? (Sim / Não) |
| What year were they created? | Vem de que ano? |
| Who created the document?  Ministry of Health  PMI  Other partner (specify: )  Not sure | Quem desenvolvou o documento?  Ministério da Saúde  Iniciativa Presidencial dos Estados Unidos  Contra a Malaria  Outro Parceiro (especifique: )  Não sabe |
| Do you have access to a copy of the dosing schedule for ASAQ? (Yes / No) | Tem acesso ao guía da dosagem de ASAQ? (Sim / Não) |
| What year were they created? | Vem de que ano? |
| Who created the document?  Ministry of Health  PMI  Other partner (specify: )  Not sure | Quem desenvolveu o documento?  Ministério da Saúde  Iniciativa Presidencial dos Estados Unidos  Contra a Malaria  Outro Parceiro (especifique: )  Não sabe |

| **Knowledge** | **Conhecimento** |
| --- | --- |
| *Now I will ask you some questions about your knowledge of malaria case management. Your answer will be kept confidential. We can discuss any doubt you might have at the end of the survey.* | *Agora vou fazer-lhe algumas perguntas sobre o conhecimento do manejo de casos de malaria. Suas respostas serão mantidas confidenciais. Podemos discutir qualquer dúvida que tiver no fim do inquérito.* |
| What is the first line treatment for uncomplicated malaria in Mozambique?  Sulfadoxine-pyrimethamine (Fansidar)  Quinine  Arthemeter-Lumefantrine (Coartem)  Artesunate  Do not know | Que e a primeira linha de tratamento para malaria nao complicada em Moçambique?  Sulfadoxina-pyrimethamina (Fansidar)  Quinino  Arteméter-Lumefantrina (AL)  Artesunato  Não sabe |
| Which of these illnesses do not cause fever?  Urinary infection  Upper respiratory infection  Diabetes  Malaria  Dengue | Qual destas doenças não causam febre?  Infeção urinaria  Infeção das vias áreas superiores  Diabetes  Malaria  Dengue |
| In what situations should malaria diagnosis always be through microscopy instead of RDT (select all that apply)?  Severe malaria suspect  Children under 5 years old  Pregnant women  Follow-up of in-patients with malaria diagnosis  Uncomplicated malaria suspect in patients who were treated with an antimalarial in the last four weeks | Em quais situações deveria sempre diagnosticar a malaria através de microscopia em vez de TDR?  Suspeita malaria grave  Crianças menores de 5 anos  Mulheres gravidas  Seguimento de doentes internados com diagnóstico de malária  Suspeita de malária não complicada em doentes que foram tratados com anti-maláricos nas últimas quatro semanas |
| When using an RDT, how long do you wait before reading the test?  5-10 minutes  10-14minutes  15-20 minutes  20-30 minutes | Ao fazer TDR, quanto tempo espera antes de fazer a leitura?  5-10 minutos  10-14 minutos  15-20 minutos  20-30 minutos |
| How many mg/kg of artesunate should you administer to an adult with severe malaria?  1.2 mg/kg  2.4 mg/kg  4.0 mg/kg | Quantos mg/kg de artesunato deve administrar ao um adulto com malaria grave?  1.2 mg/kg  2.4 mg/kg  4.0 mg/kg |

| **The following statements are true or false:**   - The best way to write laboratory results for inpatients is the traditional way with crosses. - Pregnancy is not a contra-indication for the administration of Artesunate in a pregnant woman with severe malaria - Clinical malaria is that in which a patient has the clinical symptoms suggestions of malaria, but the RDT and microscopy do not show the presence of plasmodium - There is not contraindication to the taking of Fansidar, as such every pregnant woman should receive this medicine in ANC services. - The epidemiological weeks begins on Monday and ends on Friday - Thrombocytopenia can be a symptom of severe malaria. - The NMCP receommends that all patients with severe malaria receive a dose of 20 mg/ Kg of weight of quinine (attack dose) - When conducting a rapid diagnostic test of malaria, you must wait the amount of time recommended by the manufacturer to know the definitive results even if it the result is positive in the third minute. | **As seguintes afirmações são Falsa ou Verdadeira:**   - A melhor maneira de apresentar resultados laboratoriais em doentes internados é a tradicional forma de cruzes. - A gravidez não é contra-indicação para administração de Artesunato na mulher grávida com malária grave/complicada. - Malária clínica é aquela em que o paciente apresenta quadro clínico sugestivo entretanto o TDR e HTZ não revela presença de plasmódio. - Não existe nenhuma contra-indicação para a toma do fansidar (SP), portanto toda mulher grávida deve receber este medicamento na CPN. - A semana epidemiológica inicia na segunda-feira e termina na sexta-feira. - Trombocitopénia pode ser um sinal de malária grave. - O PNCM recomenda que todos doentes graves devem receber a dose de 20mg/Kg de peso de quinino (dose de ataque). - Durante a realização de teste de diagnóstico rápido de malária, se o resultado for posítivo no terceiro minuto é obrigatório esperar o tempo recomendado pelo fabricante para a revelação do resultado definitivo. |
| --- | --- |
| **Healthcare Worker Supervision** | **Supervisão do profissional de saúde** |
| Did you have any supervisory visits in the last 6 months? (Yes / No) | Recebeu uma visita de supervisão nos últimos 6 meses? (Sim / Não) |
| How many supervisory visits did you have in the last 6 months? | Quantas visitas de supervisão recebeu nos últimos 6 meses? (Sim / Não)v |
| Who participated in the malaria supervision visits that you received in the past year?  National Ministry of Health  Provincial Ministry of Health  District Ministry of Health  Health Facility  Partner (specify: )  Do not remember | Quem participou nas visitas de supervisão de malaria no ultimo ano?  Ministério da Saúde Nacional  DPS  DDS  Unidade Sanitária  Parceiro (especifique: )  Não recorda |
| Did any of these supervisory visits include appropriate use of antimalarials? (Yes / No) | Será que alguma destas supervisões avaliou a sua gestão de casos de malária? (Sim / Não) |
| Did any of these supervisory visits include observation of patient consultations? (Yes / No) | Será que durante alguma destas supervisões o(a) supervisor(a) observou uma consulta sua? (Sim / Não) |
| Did any of these supervisory visits include provision of feedback? (Yes / No) | Será que durante alguma destas supervisões o(a) supervisor(a) deu-lhe retroinformação? (Sim / Não) |
| Which drug or drugs were you supervised on?   \| Arthemeter-Lumefantrine \| \| --- \| \| Artesunate-amodiaquine \| \| Dihidroartemisinine-piperaquine \| \| Sulfadoxine-pirimetamine \| \| Quinina pills \| \| Injectable quinine \| \| Chloroquine \| \| Primaquine \| \| Injectable artesunate \| \| Rectal artesunate \| \| Intramuscular artemether \| \| Other \| | Recebeu supervisões sobre quais medicamentos?   \| Arteméter-lumefantrina \| \| --- \| \| Artesunato-amodiaquina \| \| Dihidroartemisinina-Piperaquina \| \| Sulfadoxina-pirimetamina \| \| Quinina comprimidos \| \| Quinina injectável \| \| Cloroquina \| \| Primaquina \| \| Artesunato injectável \| \| Artesunato rectal \| \| Arteméter intramuscular \| \| Outro \| |
