# Supplementary figures and images for: Quality of malaria services offered in public health facilities in three provinces of Mozambique: a cross-sectional study
Source: Malar J. 2019 May 6;18:162. doi: 10.1186/s12936-019-2796-9 (PMC6503352; doi:10.1186/s12936-019-2796-9)

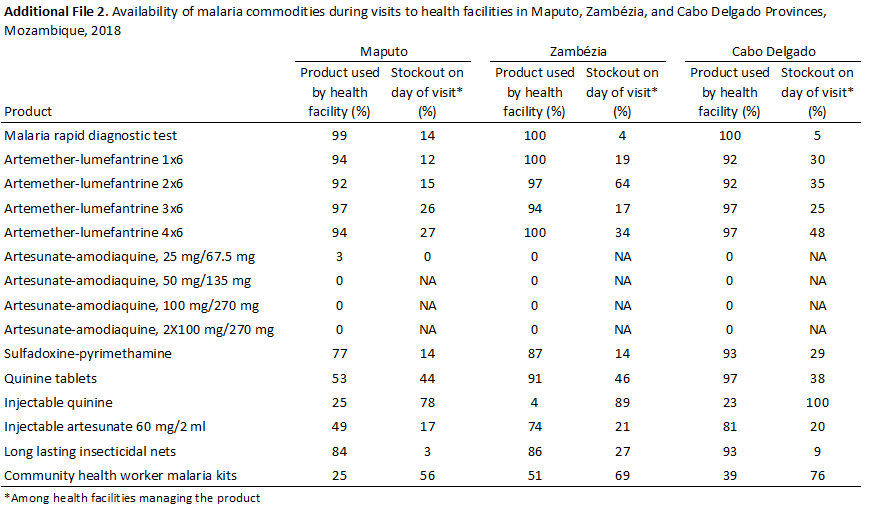

Supplement: Supplementary file 2 — Additional file 2. Availability of malaria commodities during visits to health facilities in Maputo, Zambézia, and Cabo Delgado Provinces, Mozambique, 2018. [file 12936_2019_2796_MOESM2_ESM.docx]

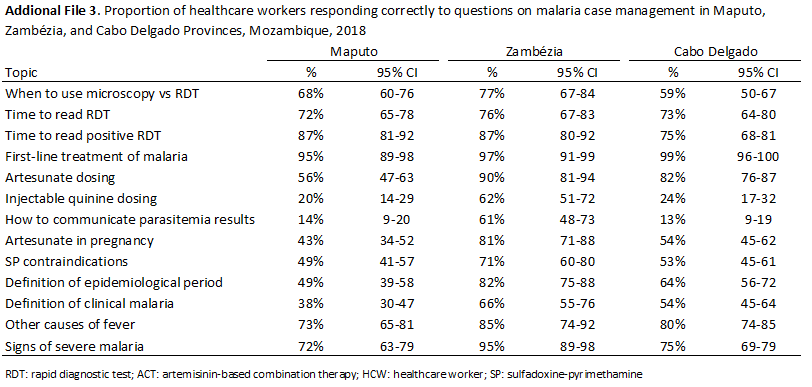

Supplement: Supplementary file 3 — Additional file 3. Proportion of healthcare workers responding correctly to questions on malaria case management in Maputo, Zambézia, and Cabo Delgado Provinces, Mozambique, 2018. [file 12936_2019_2796_MOESM3_ESM.docx]
